# The Role of Motor Inhibition During Covert Speech Production

**DOI:** 10.3389/fnhum.2022.804832

**Published:** 2022-03-09

**Authors:** Ladislas Nalborczyk, Ursula Debarnot, Marieke Longcamp, Aymeric Guillot, F-Xavier Alario

**Affiliations:** ^1^Aix Marseille Univ, CNRS, LPC, Marseille, France; ^2^Aix Marseille Univ, CNRS, LNC, Marseille, France; ^3^Inter-University Laboratory of Human Movement Biology-EA 7424, University of Lyon, University Claude Bernard Lyon 1, Villeurbanne, France; ^4^Institut Universitaire de France, Paris, France

**Keywords:** covert speech, inner speech, motor imagery, motor simulation, motor control, motor inhibition

## Abstract

Covert speech is accompanied by a subjective multisensory experience with auditory and kinaesthetic components. An influential hypothesis states that these sensory percepts result from a simulation of the corresponding motor action that relies on the same internal models recruited for the control of overt speech. This simulationist view raises the question of how it is possible to imagine speech without executing it. In this perspective, we discuss the possible role(s) played by motor inhibition during covert speech production. We suggest that considering covert speech as an inhibited form of overt speech maps naturally to the purported progressive internalization of overt speech during childhood. We further argue that the role of motor inhibition may differ widely across different forms of covert speech (e.g., condensed vs. expanded covert speech) and that considering this variety helps reconciling seemingly contradictory findings from the neuroimaging literature.

## 1. Introduction

The ability to mentally examine our verbal thoughts is central to our subjective experience. This covert (internal) production of speech typically accompanies everyday activities such as problem solving (Sokolov, 1972; Baldo et al., 2005), future planning (D'Argembeau et al., 2011), reading (e.g., Lœvenbruck et al., 2005; Perrone-Bertolotti et al., 2012), or writing (Frith, 1979). Because *overt* speech production results from sequences of motor commands that are assembled to reach a given communication goal, it belongs to the broader category of motor actions (Jeannerod, 2006a). Therefore, a parallel can be drawn between covert speech, also known as *inner speech* or *speech imagery* (for reviews, see Perrone-Bertolotti et al., 2014; Alderson-Day and Fernyhough, 2015; Lœvenbruck et al., 2018), and other imagined actions (i.e., motor imagery). The motor simulation theory of motor imagery (Jeannerod, 1994, 2001, 2006b) postulates a continuum between the covert and the overt execution of an action, and that action representations can operate off-line via a simulation mechanism.

However, the proposal that overt and covert actions share common processes and neural circuits is faced with a serious problem. If the neural circuits used for the control of overt actions are also used for covert actions, how can covert actions not lead to execution? This puzzle was coined as *the problem of inhibition of execution* by Jeannerod (2001). In this perspective, we examine some theoretical and experimental consequences that emerge from considering covert speech as inhibited overt speech. First, we explore the role and plausible neural implementation of inhibitory mechanisms during covert speech production. Second, we relate the maturation of inhibitory control during childhood with the progressive internalization of overt speech. Third, we consider how inhibitory mechanisms may play different roles across different forms of covert speech. By bridging recent results from the covert speech, motor imagery, and motor inhibition literature, we highlight some novel and possibly fruitful lines of research.

## 2. Covert Speech Production as Inhibited Overt Speech Production

### 2.1. Cognitive and Neural Mechanisms Supporting Motor Inhibition

First and foremost, we need to make a distinction between at least two different types of inhibition. First, cognitive inhibition, defined as the stopping or overriding of a mental process, with or without intention (MacLeod, 2007). Second, the inhibition of physical response, or motor inhibition, defined broadly as the withholding, suppression, or overriding of an inappropriate, prepotent, or unwanted motor response (Aron, 2007; O'Shea and Moran, 2018). Here, we are concerned with the latter. Ridderinkhof et al. (2014) further described the concept of response inhibition on three continuous dimensions: intentionality, premeditation, and specificity. Inhibition can be employed with more or less intentionality, planned ahead or employed in the moment, and applied to a specific action and effector, or more globally, to all actions, and/or effectors.

Within Ridderinkhof et al.'s classification of response inhibitions, we hypothesize that covert speech involves an intentional (we know we want to produce these actions covertly rather than overtly) but implicit/automatic (we do not explicitly think about not producing movements) and planned ahead form of response inhibition. The distinction between implicit and explicit inhibition seems important to highlight. The type of motor inhibition that may be at play during motor imagery is still different from the “proactive inhibition” in the motor inhibition literature. Indeed, in behavioral tasks aiming to assess proactive inhibition, participants are instructed not to execute an action. In contrast, while doing motor imagery, participants are asked to imagine the action, which indirectly implies that it should not be executed overtly (Guillot et al., 2012). Moreover, the type of motor inhibition that is implemented during covert speech necessarily has to be planned ahead, otherwise speech acts would sometimes be (at least partially) executed. Finally, the level at which motor inhibition may be applied can be inferred from the example of hand movements. Rieger et al. (2017) used an action mode (overt vs. covert) switching paradigm, to show that the motor imagery of hand movements is accompanied by both global and effector-specific inhibition (these results were also replicated in Scheil and Liefooghe, 2018; Bart et al., 2021a,b,c). Here, we hypothesize that inhibition during covert speech may similarly apply both globally and in an effector-specific manner.

Based on evidence from electrophysiological, neuroimaging, and clinical studies, Guillot et al. (2012) suggested several possible routes whereby motor commands can be inhibited during motor imagery. First, cerebral regions such as the pre-supplementary motor area (pre-SMA) (Kasess et al., 2008) or the right inferior frontal gyrus (rIFG) may weaken the motor commands that are emitted during motor imagery (e.g., Angelini et al., 2015, 2016). More precisely, the pre-SMA and the rIFG may work together to intercept the action process via the basal ganglia (subthalamic nucleus), hence suppressing the output from the basal ganglia which in turn might inhibit the primary motor cortex (Aron, 2011). Second, motor imagery has been shown to be associated with modulations of short-interval intracortical inhibition within the primary motor cortex itself (Neige et al., 2020). Third, downstream regions in the cerebellum (e.g., Lotze et al., 1999), in the brainstem (e.g., Jeannerod, 2001, 2006a), or at the spinal level may contribute to motor inhibition at a later stage.

In addition to these three possible routes, another possibility highlighted by Guillot et al. (2012) is that motor inhibition can be integrated within the representation of the action to be produced internally, so that only subthreshold motor commands may be involved during motor imagery (hereafter referred to as the “subliminal level hypothesis,” see also Glover et al., 2020; Bach et al., 2021). It has been suggested that during covert speech production, motor commands would be “simply specified in subthreshold way, requiring no active inhibition” (Geva, 2018). However, stating that covert speech (or motor imagery, more generally) only involves subthreshold activity (and therefore is not accompanied by the emission of motor commands that are inhibited) simply shifts the problem from “how and where motor commands are subsequently inhibited” to “how and where the magnitude of activity in the motor system is planned or monitored” (see also Scheil and Liefooghe, 2018). In other words, we still need to explain how (in a mechanistic and/or developmental way) this activity is maintained at a subthreshold level. In this section, we provided empirical arguments in favor of the “active inhibition hypothesis.” Proponents of the “subliminal level hypothesis” need to clarify how this activity is maintained at a subthreshold level during covert speech production, thus preventing execution.

The putative involvement and functional role of (cortical and subcortical) inhibitory mechanisms during covert speech could be assessed in several ways. First, it could be assessed by experimentally manipulating the activity of the inhibitory network responsible for preventing execution during motor imagery. For instance, transcranial magnetic stimulation (TMS) could be used to interrupt these inhibitory mechanisms and thus trigger execution during motor imagery. Second, it could be assessed by looking at covert speech production in patients with acquired (focal) brain damage. For instance, Schwoebel et al. (2002) observed that bilateral parietal lesions can lead patients to execute actions when they asked to imagine them, suggesting a failure of inhibitory mechanisms. Third, the role of inhibitory mechanisms during covert verbal actions could be examined in populations with well-identified inhibitory deficits. For instance, Tourette syndrome is a childhood-onset neurological disorder affecting approximately 1% of children and characterized by chronic motor and phonic tics (Jackson et al., 2015). Verbal tics can consist of repeating sounds, words, or utterances (palilalia), producing inappropriate or obscene utterances (coprolalia), or the repetition of another's words (echolalia). In their review, Jackson et al. (2015) suggested that increased control over motor outputs, acquired by repeatedly trying to suppress tics, is brought about by local increases in GABAergic “tonic” inhibition within regions such as the SMA, leading to localized reductions in the gain of motor excitability. For these reasons, comparing the neural implementation of inhibitory mechanisms during covert speech in patients with Tourette syndrome and healthy controls may shed light on the role and flexibility of these mechanisms.

### 2.2. Covert Speech Development: Learning Not to Produce Speech

Watson (1919) suggested that thought was rooted in overt speech. In his terminology, thought referred to covert speech. Hence, his view was that covert speech matures from overt speech. Vygotsky (1934) further elaborated the idea that covert speech is internalized during childhood from private egocentric speech, that is, from self-addressed overt speech. Fernyhough (2004) extended these ideas by proposing four levels of internalization: external dialogue, private speech, expanded inner speech, and condensed inner speech. These levels represent stages of development but also define movements between levels, that is, how a speaker may transform overt speech to covert speech, and conversely. The level at which speech is expressed may depend on inhibitory control applied at different levels in the production flow, such as the formulation or the articulatory planning level (Grandchamp et al., 2019). Therefore, producing covert speech crucially depends on successfully inhibiting speech production at several levels.

Here, we hypothesize that the progressive internalization of speech during childhood may be related to the development of inhibitory abilities. This hypothesis could be tested in several ways. First, the relation between speech internalization and inhibitory abilities could be assessed during development at the critical ages (i.e., between 6 and 8 years). We would expect the ability to imagine actions, and speech specifically, to be positively correlated with motor inhibition at this age. Wang et al. (2021) provided correlational evidence that motor imagery (assessed in a hand laterality judgement task) and motor inhibition performance (assessed in a stop-signal task) improve together between 7 and 11 years old, and that these two abilities correlate at 7 years old but did not correlate at 11 years old. This suggests that inhibitory control may play a more prominent role when speech is being internalized, but its role may weaken with expertise. This would be consistent with results from training studies suggesting that, with growing expertise, mental imagery increasingly relies on memory-based processes (e.g., Jolicoeur, 1985; Tarr and Pinker, 1989).

Second, the hypothesized co-development of motor imagery and response inhibition abilities could be tested by examining how novel actions are internalized in adults. Consider for instance how the act of producing speech can be paralleled with the act of playing a music instrument (e.g., the piano). Both actions consist in the coordination of complex movements that result in some modifications of the environment, that in turn generate sensory feedback (e.g., kinaesthetic, auditory) for the agent. This analogy suggests that we might be able to study the development of internal models responsible for the sensory experience accompanying imagined actions in the adult mind (e.g., when an individual is learning either a novel music instrument or a new language with speech sounds that are not present in his/her native language). By examining the development of novel imagined actions in the adult mind and by using motor interference (e.g., articulatory suppression) procedures, we might gain new insights about the internalization of speech during childhood^1^.

### 2.3. Does Covert Speech Always Involve Motor Inhibition?

The production of covert speech is often, although not always and not for everyone, accompanied by the feeling of *hearing* speech (Hurlburt, 2011). However, covert speech may also be accompanied by the feeling of *producing* speech. These two facets of covert speech are characterized by different phenomenological experiences. In this section, we discuss how these two forms of covert speech may require motor inhibition to a different extent.

The dual stream prediction model (Tian and Poeppel, 2012, 2013; Tian et al., 2016) describes two neural pathways that may provide the auditory content of covert speech. First, the simulation-estimation prediction stream implements a motor-to-sensory transformation via motor simulation, that is, by simulating speech movements and the perceptual changes that would be associated with these movements (see also Lœvenbruck et al., 2018, for a similar proposal). This stream includes cerebral areas involved in speech motor preparation such as the supplementary motor area, the inferior frontal gyrus, the premotor cortex, and the insula, as well as brain areas involved in somatosensory estimation and perception such as primary and secondary somatosensory regions, the parietal operculum, and the supramarginal gyrus (Tian et al., 2016). Second, the memory-retrieval prediction stream provides auditory percepts by “reconstructing stored perceptual information in modality-specific cortices” (Tian et al., 2016). This mechanism provides sensory percepts without the need for computing the predicted sensory consequences of (non executed) motor commands. Auditory percepts may be retrieved from various memory sources, relying (amongst others) on the hippocampal formation (Tian et al., 2016), or from a broad fronto-temporo-parietal lexico-semantic network (for more details, see Tian et al., 2016).

The balance between the mechanisms of simulation and memory retrieval may depend on the circumstances promoting covert speech or, in the lab, on the precise instructions given to participants, which may cue them to produce different forms of covert speech. For instance, either one of these two streams may be preferentially recruited depending on whether participants are instructed to “imagine speaking” or to “imagine hearing” (see also the distinction between the “inner ear” and the “inner voice,” e.g., Smith et al., 1992). In line with this hypothesis, Tian et al. (2016) have shown that inner speaking recruits brain regions in the simulation stream more strongly than inner hearing, which conversely recruits more strongly brain regions in the memory-retrieval stream. Ma and Tian (2019) have shown that inner speaking and inner hearing have distinct magnetoencephalographic (MEG) correlates and distinct effects on a subsequent phonetic categorization task (discriminating /ba/ vs. /da/).

In line with Tian and Poeppel (2012), we suggest that the balance between these two mechanisms may also depend on a participant's situational (e.g., surrounding noise) and individual (e.g., expertise) characteristics. We further suggest that a common currency to determine the recruitment of either one of these mechanisms is the computational cost of (or equivalently, the computational resources available for) each alternative. To clarify, we borrow the concept of memoization as applied to cognition and mental imagery by Dasgupta and Gershman (2021) (cf. Box 1). In these authors' view, memory can be considered as a computational resource that facilitates computational reuse through memoization. In the context of motor and speech imagery, memoization can be seen in the increasing reliance on memory in the course of learning.

Box 1 **Memoization**

Memoization is a programming technique used to speed-up algorithms or programs. It avoids redundant computation by storing computational results and reusing them later (Dasgupta and Gershman, 2021). When calling a function (where a function can be a motor primitive), the function call is intercepted by a *memoizer* that inspects the previous calls of a function and its outputs. If a function has already been called with the same input, then the previously computed output is retrieved and reused.

In the context of covert speech, memoization can be postulated as the process by which covert speech percepts produced by motor simulation are stored for later retrieval and use without invoking the motor simulation mechanism.

In other words, situational (extrinsic) and individual (intrinsic) characteristics jointly determine the computational cost of (or equivalently, the available computational resources for) the task, which in turn determines the balance between the simulation and association mechanisms. For instance, we hypothesize that novel and/or difficult tasks (which are both computationally more expensive, ceteris paribus) may rely more on the simulation mechanism, whereas well known and/or easy tasks may rely more on associative mechanisms. This idea is supported by several studies showing a greater increase in facial EMG activity during the reading of difficult text or while performing difficult mental arithmetic tasks, compared to easier tasks (e.g., Faaborg-Andersen et al., 1958; Sokolov, 1972), suggesting a greater involvement of the speech motor system. Alternatively, these results may suggest a lesser involvement of inhibitory mechanisms (see also the discussion in Nalborczyk, 2019, 2020). This is congruent with the increased reliance on associative mechanisms with greater expertise, as discussed previously.

To sum up, whereas inner speaking may involve active inhibition of motor commands, inner hearing may not. These disparities between inner speaking and inner hearing may explain the variety of neural correlates reported for covert speech production (as reviewed for instance in Geva, 2018). More generally, different forms of covert speech may vary in condensation (from thinking without words to thinking in words), dialogicality (whether covert speech features monologes or dialogues), or intentionality (for more details, see Grandchamp et al., 2019) and may thus require inhibitory control to a different extent, from no inhibition at all for condensed forms of covert speech to active inhibition of motor commands for fully expanded forms of covert speech.

## 3. Conclusions

We explored some of the theoretical and experimental consequences that emerge from considering covert speech production as an inhibited form of overt speech production. To this end, we connected results from the motor imagery, motor inhibition, and covert speech domains. Regarding the role and implementation of general-purpose inhibitory mechanisms during the production of covert speech, we suggested that these may be similar to the inhibitory network responsible for proactive response inhibition and we summarized some propositions from this literature. We related the development of response inhibition abilities in childhood development with the purported internalization of private speech around the same period. From the response inhibition perspective, the internalization of speech from overt to covert speech may essentially be considered as “learning not to execute speech.”

Regarding the neural origin of the sensory experience of covert speech, we discussed the dual stream prediction model (Tian and Poeppel, 2012, 2013; Tian et al., 2016), which suggests that these sensory percepts may be provided either by a motor-simulation process or by a memory-retrieval process. We suggested that the balance between these two mechanisms may be determined by task instructions, which may prompt different forms of covert speech, and also by the computational cost of the task. More precisely, novel or more difficult tasks are expected to rely more on the motor-simulation mechanisms whereas well-known and/or easy tasks may rely more on a “memoized version” of the motor simulation: the memory-retrieval prediction stream. Whereas the former mechanism should involve active inhibitory mechanisms, the latter should not, as there should be no (or less) motor commands to inhibit.

These propositions pave the way for several lines of research that should consolidate our understanding of the relations between overt and covert speech production. Several outstanding questions remain. Amongst others, further research should aim at testing whether and how the development of inhibitory control relates with the progressive internalization of speech during childhood. Do individual and situational constraints shape the role of motor inhibition during covert speech production? Is covert speech affected by poor or degraded inhibitory control? Can we experimentally force the externalization of speech in adults, for example through neurostimulation? The use of neurostimulation and the comparison between healthy controls and patients with well-identified inhibitory deficits could help refine the involvement of these inhibitory mechanisms during covert speech production, which may lead to applied outcomes in the care of motor and verbal tics.

## Data Availability Statement

No data were used in this paper. However, the source code is available at https://osf.io/dsfgb/.

## Author Contributions

All authors: conceptualization and writing—review and editing. LN, ML, F-XA: funding acquisition. UD, ML, AG, and F-XA: supervision. LN: writing—original draft. All authors contributed to the article and approved the submitted version.

## Funding

This work was carried out within the Institut Convergence ILCB (ANR-16-CONV-0002), has benefited from support from the French government, managed by the French National Agency for Research (ANR) and the Excellence Initiative of Aix-Marseille University (A*MIDEX).

## Conflict of Interest

The authors declare that the research was conducted in the absence of any commercial or financial relationships that could be construed as a potential conflict of interest.

## Publisher's Note

All claims expressed in this article are solely those of the authors and do not necessarily represent those of their affiliated organizations, or those of the publisher, the editors and the reviewers. Any product that may be evaluated in this article, or claim that may be made by its manufacturer, is not guaranteed or endorsed by the publisher.

## Footnotes

^1^We should keep in mind the obvious limitation that the child mind is not equivalent to the adult mind, nor is it equivalent to a smaller version of the adult mind. Nevertheless, examining the development of novel imagined actions in adults avoids the contamination of the process of interest (imagined action) by developmental confounds.
